# Butyrate attenuates lipolysis in adipocytes co-cultured with macrophages through non-prostaglandin E2–mediated and prostaglandin E2–mediated pathways

**DOI:** 10.1186/s12944-016-0387-0

**Published:** 2016-12-09

**Authors:** Hideo Ohira, Wao Tsutsui, Rie Mamoto, Sayaka Yamaguchi, Masako Nishida, Miki Ito, Yoshio Fujioka

**Affiliations:** Faculty of Nutrition, Kobe Gakuin University, 518 Arise, Ikawadani-cho, Nishi-ku, Kobe, 6512180 Japan

**Keywords:** Butyrate, Adipocyte, Macrophage, Lipolysis, Prostaglandin E2

## Abstract

**Background:**

Interactions between adipocytes and macrophages are associated with metabolic disorders. Production of pro-inflammatory mediators and the release of free fatty acids (FFAs) increase when these cells are co-cultured; butyrate significantly diminishes these effects by suppressing both the macrophage inflammatory and adipocyte lipolysis pathways. Butyrate is known to up-regulate the expression of prostaglandin E2 (PGE2). Therefore, we hypothesized that PGE2 is associated with the suppression of lipolysis by butyrate in co-culture.

**Methods:**

Using contact or transwell co-culture methods with differentiated 3T3-L1 adipocytes and RAW264.7 macrophages, we investigated the effects of butyrate on the release of PGE2 into the medium and on lipolysis in adipocytes. To elucidate the underlying mechanism, we examined the effects of butyrate on cyclooxygenase-2 (COX2) and phospholipase A2 (PLA2) in co-cultured cells, and cyclic adenine monophosphate (cAMP) and protein kinase A type 1-α regulatory subunit (PRKAR1A) in co-cultured adipocytes. Silent interfering (si)RNA targeting of G-protein–coupled receptor (GPR)41 and 109A was employed to examine the effect on lipolysis in TNF-α–stimulated adipocytes.

**Results:**

Co-culture increased PGE2 release into the medium, compared with cells cultured separately. Butyrate significantly increased PGE2 production. Co-culture elevated COX2 expression in macrophages and adipocytes, and butyrate further enhanced this effect. Co-culture enhanced cytosolic PLA2 activity in macrophages, which was further enhanced by butyrate. As for lipolysis, co-culture increased the release of FFAs and free glycerol into the medium, whereas butyrate (and to a lesser extent, PGE2) suppressed FFAs and free glycerol release. An inhibition study using a prostaglandin E receptor 3–selective antagonist suggested that approximately 40% of the suppressive effect of butyrate depends on the PGE2-mediated pathway, whereas 60% depends on a non-PGE2–mediated pathway. Co-culture increased cAMP and PRKAR1A levels in adipocytes, whereas butyrate restored the levels to those of the control. Similarly, in TNF-α–stimulated adipocytes, butyrate reduced FFAs and free glycerol release. siRNA inhibition of GPR41 and GPR109A suggested that the GPR109A-mediated pathway predominates, but the GPR41-mediated pathway also regulates the effect of butyrate on lipolysis in TNF-α–stimulated 3T3-L1 cells.

**Conclusions:**

Butyrate attenuates lipolysis in adipocytes co-cultured with macrophages via non-PGE2–mediated and PGE2-mediated pathways.

## Background

Recent studies have demonstrated a strong relationship between obesity (particularly visceral fat accumulation) and the development of cardiovascular diseases [1, 2]. Products derived from interactions between adipocytes and macrophages that may cause metabolic disorders leading to the formation of atherosclerotic lesions include cytokines, chemokines, and adipokines [3–5]. Suganami et al. proposed that the paracrine loop involving free fatty acids (FFAs) derived from adipocytes and monocyte chemoattractant protein-1 (MCP-1) and tumor necrosis factor-α (TNF-α) produced by macrophages form a vicious cycle that accelerates hypertrophy of adipocytes [6].

Recently, we confirmed that co-culturing adipocytes and macrophages results in a marked up-regulation of the expression of various pro-inflammatory mediators, including TNF-α, MCP-1, and interleukin (IL)-6. In addition, butyrate significantly reduces the expression of these mediators by suppressing both the macrophage inflammatory and adipocyte lipolysis pathways [7].

Prostaglandin E2 (PGE2), an adipocyte and macrophage product, is known to be involved in a variety of cell signaling pathways [8–10]. Production of PGE2 requires a supply of arachidonic acid derived from phospholipids released by phospholipase A2 (PLA2) to serve as the substrate for cyclooxygenase-2 (COX2) [8–10]. Short-chain fatty acids (SCFAs), such as acetic acid, propionic acid, and butyric acid, are produced by anaerobic bacterial fermentation of undigested carbohydrates in the colon [11] and are rapidly absorbed to provide energy for the colorectal epithelium. Levels of SCFAs are increased in rectal mucosal blood [12]. The SCFA sodium butyrate (butyrate) modulates the production of inflammatory mediators [13–15]. Reports have demonstrated that butyrate up-regulates the expression of PLA2, COX2, and PGE2 in Kupffer cells [16] and COX2 and PGE2 in human peripheral blood mononuclear cells [17].

Whether PGE2 is associated with the suppressive role of butyrate on the vicious cycle is an important question. In order to clarify the anti-lipolytic effect of butyrate in the interactions between macrophages and adipocytes, in the present study, we examined the effect of butyrate on the production of PGE2 and the expression of PLA2 and COX2. We also examined the effect of butyrate on components of the lypolytic process, including the TNF-α/nuclear factor-kappa B (NF-κB) pathway and PGE2-associated signaling factors such as cyclic adenosine monophosphate (cAMP) and protein kinase A type 1-α regulatory subunit (PRKAR1A), in co-cultured adipocytes.

## Results

### Effect of butyrate on PGE2 production in co-cultured cells

Preliminary experiments using the MTT assay confirmed that incubating RAW264.7 cells with >2.0 mmol/L butyrate and 3T3-L1 cells with >10.0 mmol/L butyrate for 24 h resulted in excessive toxicity (data not shown). Therefore, the maximum concentration of butyrate used in all subsequent experiments was 1.0 mmol/L.

**Fig. 1:**
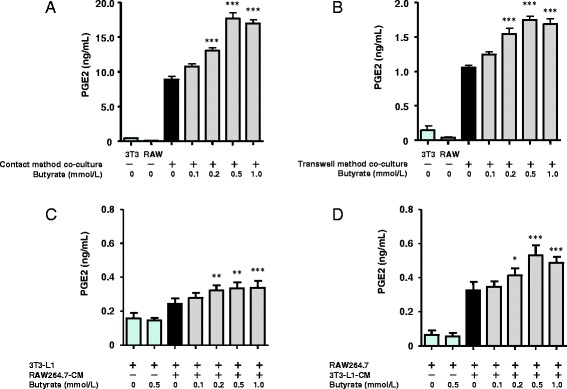
Effect of butyrate on the production of PGE2 induced in cells co-cultured using the contact method for 24 h (**a**), in cells co-cultured using the transwell method for 24 h (**b**), in 3T3-L1 cells incubated with RAW264.7-CM for 24 h (**c**), and in RAW264.7 cells incubated with 3T3-L1-CM for 24 h (**d**). The concentration of PGE2 in the co-culture medium was determined by ELISA. The concentration of PGE2 produced by non–co-cultured 3T3-L1 or RAW 264.7 cells served as the control. Values from five independent experiments are expressed as the mean ± SD. *p < 0.05, **p < 0.01, and ***p < 0.001 versus co-culture, as determined by ANOVA and the Tukey-Kramer test

We first examined the effect of butyrate on PGE2 production. The concentration of PGE2 in the co-culture medium increased markedly when cells were cultured using the contact method, whereas the concentration was very low when cells were cultured separately (Fig. 1a). Production of PGE2 by cells co-cultured using the contact method in the presence of butyrate increased significantly in a dose-dependent manner up to a butyrate concentration of 0.5 mmol/L. A similar effect of butyrate was observed in cells co-cultured using the transwell method, although the peak PGE2 value was lower than that produced by cells co-cultured using the contact method (Fig. 1b). When cultured separately, 3T3-L1 cells produced more PGE2 than RAW264.7 cells (Fig. 1a, b). To examine which cells are the predominant PGE2 producers under co-culture conditions with and without butyrate, we examined PGE2 production in cells cultured separately in the presence of conditioned medium (CM) of the other cell type. 3T3-L1 cells produced more PGE2 when cultured in RAW264.7-CM, and butyrate significantly increased PGE2 production, by approximately 2-fold compared with cells cultured separately (Fig. 1c). Similarly, RAW264.7 cells produced more PGE2 when cultured in 3T3-L1-CM; the addition of butyrate significantly increased PGE2 production, by approximately 8-fold compared with cells cultured separately (Fig. 1d). Thus, both cell types produce PGE2, and the presence of butyrate under co-culture conditions stimulates PGE2 production, particularly in RAW264.7 cells.

### Effect of pertussis toxin (PTX) on PGE2 production in co-cultured cells

**Fig. 2:**
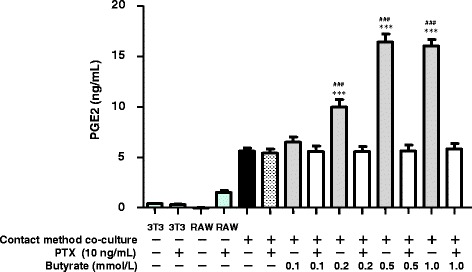
Effect of pertussis toxin (PTX) on the production of PGE2 induced by 24 h of co-culture using the contact method. The concentration of PGE2 in the co-culture medium was determined by ELISA. The concentration of PGE2 in the medium of non–co-cultured 3T3-L1 or RAW 264.7 cells served as the control. Values from five independent experiments are expressed as the mean ± SD. ***p < 0.001 versus co-culture, and ^###^ *p* < 0.001 versus co-culture with PTX, as determined by ANOVA and the Tukey-Kramer test

Previously, we found that PTX, a Gi signaling pathway inhibitor, blocks the anti-lipolytic effect of butyrate in co-cultured 3T3-L1 cells by blocking G-protein coupled receptor (GPR) 41-mediated signaling [7]. Here, as expected, PTX completely suppressed the up-regulation of PGE2 production by cells co-cultured using the contact method with ≥0.2 mmol/L butyrate (Fig. 2). GPR109A, a nicotinic acid receptor regulated by Gi signaling pathways, was recently identified as a receptor for SCFAs [18, 19]. These data suggest that butyrate may stimulate PGE2 production via GPR41- and/or GPR109A-mediated pathways.

### COX2 expression in co-cultured cells

**Fig. 3:**
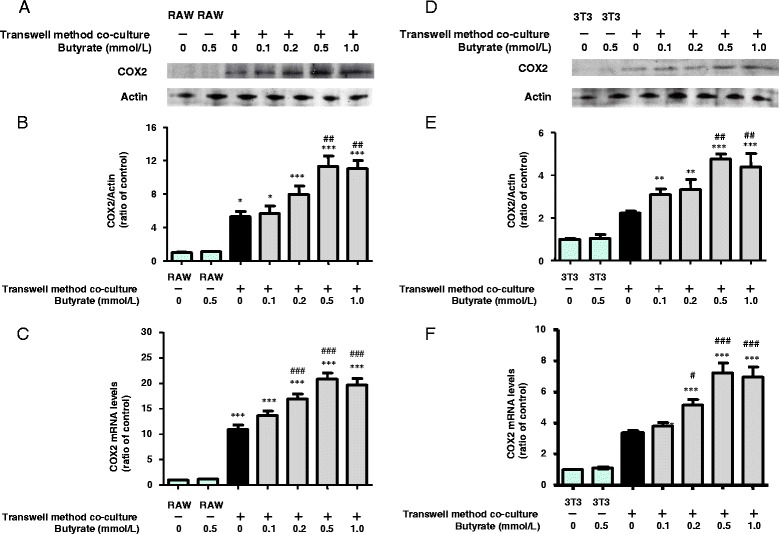
Effects of butyrate on COX2 protein and mRNA expression in RAW264.7 (**a**, **b**, **c**) and 3T3-L1 (**d**, **e**, **f**) cells co-cultured using the transwell method for 24 h. COX2 and actin proteins were detected by western blotting (**a**, **d**). The expression ratio of COX2 to actin was determined based on quantification of the relative density of western blotting bands (**b**, **e**). COX2 and β-actin mRNAs were detected using qRT-PCR (**c**, **f**), as described in the Methods. For western blotting and protein expression, *n* = 3, and for qRT-PCR, *n* = 5. Values are expressed as the mean ± SD. *p < 0.05, **p < 0.01, and ***p < 0.001 versus the control, and ^#^ *p* < 0.05, ^##^ *p* < 0.01, and ^###^ *p* < 0.001 versus co-culture, as determined by ANOVA and the Tukey-Kramer test

To clarify the mechanism by which butyrate enhances the production of PGE2, we monitored the expression of COX2 in RAW264.7 cells co-cultured using the transwell method. Although the levels of COX2 protein and mRNA expression in control and butyrate-treated RAW264.7 cells cultured separately were very low, butyrate did induce a marked and significant dose-dependent increase in COX2 protein and mRNA expression in co-cultured RAW264.7 cells (Fig. 3a-c).

Similar butyrate-induced increases in the expression of COX2 protein and mRNA were observed in co-cultured 3T3-L1 cells (Fig. 3d-f). Thus, increased COX2 expression induced by butyrate may up-regulate PGE2 production in both RAW264.7 and 3T3-L1 cells in co-culture.

### Activities of calcium-dependent cytosolic PLA2 (cPLA2) and secretory PLA2 (sPLA2) in co-cultured cells

**Fig. 4:**
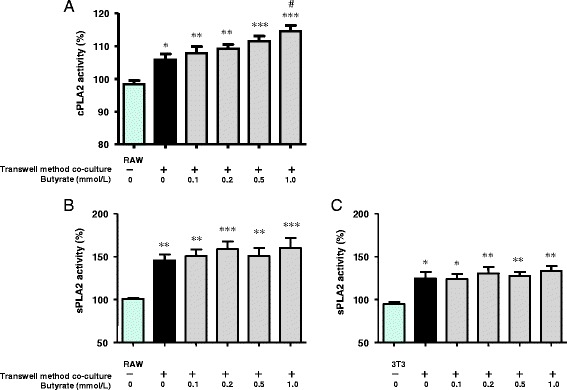
Effects of butyrate on cPLA2 activity in RAW264.7 cells (**a**) and sPLA2 activity in RAW264.7 (**b**) and 3T3-L1 (**c**) cells co-cultured using the transwell method for 24 h. The activities of intracellular cPLA2 and sPLA2 were measured using an assay kit, as described in the Methods. As the control, activity was measured in non–co-cultured cells. Values from five independent experiments are expressed as mean ± SD. *p < 0.05, **p < 0.01, and ***p < 0.001 versus the control, and ^#^ *p* < 0.05 versus co-culture, as determined by ANOVA and the Tukey-Kramer test

Next, we examined whether butyrate treatment increases the activities of cPLA2 and sPLA2 in RAW264.7 and 3T3-L1 cells co-cultured using the transwell method. Butyrate induced a significant dose-dependent increase in cPLA2 activity in co-cultured RAW264.7 cells (Fig. 4a), whereas no cPLA2 activity was detected in co-cultured 3T3-L1 cells (data not shown). The activity of sPLA2 increased in both RAW264.7 and 3T3-L1 cells co-cultured using the transwell method. However, butyrate did not enhance the sPLA2 activity in either cell type in co-culture (Fig. 4b, c).

### Adipose-specific PLA2 (AdPLA) expression in co-cultured 3T3-L1 cells

**Fig. 5:**
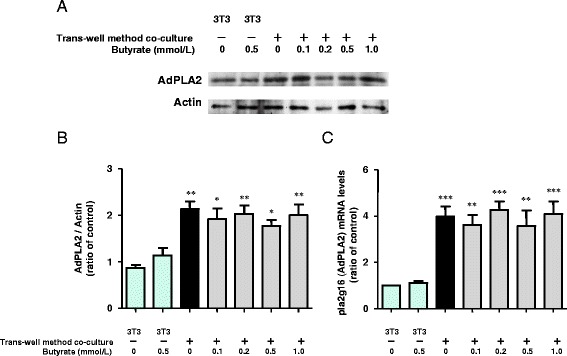
Effects of butyrate on AdPLA protein (**a**, **b**) and mRNA (**c**) expression in 3T3-L1 cells co-cultured using the transwell method for 24 h. AdPLA and actin proteins were detected by western blotting (**a**). The expression ratio of AdPLA to actin was determined based on quantification of the relative density of western blotting bands (**b**). Ala2g16 and β-actin mRNAs were detected using qRT-PCR (C), as described in the Methods. For western blotting and protein expression, *n* = 3, and for qRT-PCR, *n* = 5. Values are expressed as the mean ± SD. *p < 0.05, and **p < 0.01, ***p < 0.001 versus the control, as determined by ANOVA and the Tukey-Kramer test

AdPLA2, which was recently discovered, is highly expressed only in adipocytes, where it catalyzes the release of sn-2 fatty acids from phosphatidylcholine [10]. In the present study, we examined the expression of AdPLA2 in 3T3-L1 cells. The expression of AdPLA2 protein and mRNA were enhanced in cells co-cultured using the transwell method compared with cells cultured separately (Fig. 5a-c). Butyrate treatment had no additional effect on AdPLA2 protein and mRNA expression in either co-cultured 3T3-L1 cells or cells cultured separately. These results indicate that co-culture enhances AdPLA2 expression, but butyrate has no effect on the expression of AdPLA2 protein or mRNA.

### Effect of trichostatin A (TSA) on PGE2 production in co-cultured cells

**Fig. 6:**
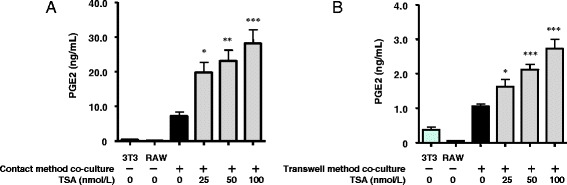
Effect of TSA on the production of PGE2 induced in cells co-cultured using the contact method for 24 h (**a**) and in cells co-cultured using the transwell method for 24 h (**b**). The concentration of PGE2 in the co-culture medium was determined by ELISA. The concentration of PGE2 produced by non–co-cultured 3T3-L1 or RAW 264.7 cells served as the control. Values from five independent experiments are expressed as the mean ± SD. *p < 0.05, **p < 0.01, and ***p < 0.001 versus co-culture, as determined by ANOVA and the Tukey-Kramer test

Butyrate is known to modulate gene transcription by inducing hyperacetylation of histones through inhibition of histone deacetylation (HDAC) [20]. To elucidate the underlying mechanism of butyrate-induced up-regulation of PGE2 production, we examined whether TSA, a HDAC inhibitor, can mimic the effect of butyrate on PGE2 production. Production of PGE2 by cells co-cultured using both the contact and transwell methods in the presence of TSA (25–100 nmol/L) increased significantly in a dose-dependent manner (Fig. 6a, b **)**. Thus, inhibition of HDAC may be one mechanism by which butyrate increases the production of PGE2.

### Effect of butyrate and prostaglandin E receptor 3 (EP3)-selective antagonist on lipolysis in co-cultured 3T3-L1 cells

Previously, we showed that butyrate significantly reduces the release of FFAs and free glycerol by diminishing total lipase activity, including that of hormone-sensitive lipase (HSL) and adipose triglyceride lipase (ATGL), in co-cultured 3T3-L1 cells [7]. In this study, we wanted to clarify whether butyrate-associated enhanced PGE2 production reduces lipolysis. PGE2 binds 4 cognate receptors, designated EP1 through EP4. Compared with other EP receptors in adipose tissue, Gαi-coupled EP3 is reportedly expressed at >10-fold markedly higher levels [10]. After binding to EP3, PGE2 down-regulates lipolysis by inhibiting cAMP production in adipocytes [10, 21]. We therefore examined whether treatment with the EP3-selective antagonist L798106 could attenuate the PGE2- and butyrate-dependent inhibition of lipolysis in adipocytes.

**Fig. 7:**
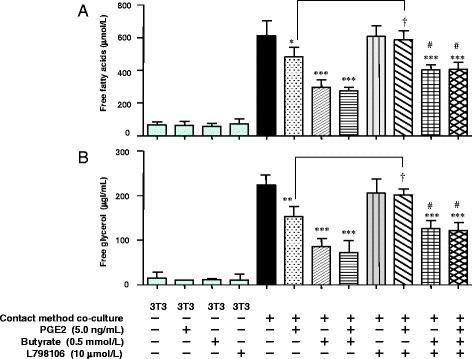
Effect of butyrate with PGE2 and/or EP3 receptor antagonist (L798106) on lipolysis in cells co-cultured using the contact method for 24 h. Concentrations of FFAs (**a**) and free glycerol (**b**) in the co-culture medium were determining using an assay kit. As the control, the concentrations of FFAs and free glycerol were determined in the medium of untreated and non–co-cultured 3T3-L1 cells. Values from five independent experiments are expressed as the mean ± SD. *p < 0.05, **p < 0.01 and ***p < 0.001 versus co-culture, and ^#^ *p* < 0.05 versus co-culture with butyrate (0.5 mmol/L), as determined by ANOVA and the Tukey-Kramer test; ^†^ *p* < 0.05 for co-culture with PGE2 (+) and L798106 (−) versus co-culture with PGE2 (+) and L798106 (+), as determined by paired *t*-test

The release of both FFAs and free glycerol into the medium was increased in cells co-cultured using the contact method. Butyrate suppressed FFAs and free glycerol release from co-cultured cells (by approximately 50% of the level observed in cells co-cultured using the contact method); exogenous PGE2 also suppressed FFAs and free glycerol release, although to a lesser extent (by approximately 20% of the level observed in cells co-cultured using the contact method) (Fig. 7a, b). Exogenous PGE2 had no significant additional suppressive effect on butyrate-treated cells co-cultured using the contact method. These results suggest that approximately 40% of the effect of butyrate depends on the PGE2-mediated pathway. The EP3-selective antagonist L798106 completely reversed the increase in FFAs and free glycerol release from exogenous PGE2-treated cells co-cultured using the contact method. Addition of L798106 with butyrate significantly suppressed FFAs and free glycerol release (by approximately 35% of the level observed in cells co-cultured using the contact method). This result suggests that approximately 60% of the effect of butyrate depends on the non-PGE2–mediated pathway. Thus, PGE2 inhibits lipolysis via EP3, and butyrate inhibits lipolysis through a mechanism involving both PGE2-mediated and non-PGE2–mediated pathways.

### Effects of PGE2 and butyrate on cAMP levels and lipolysis in co-cultured 3T3-L1 cells

**Fig. 8:**
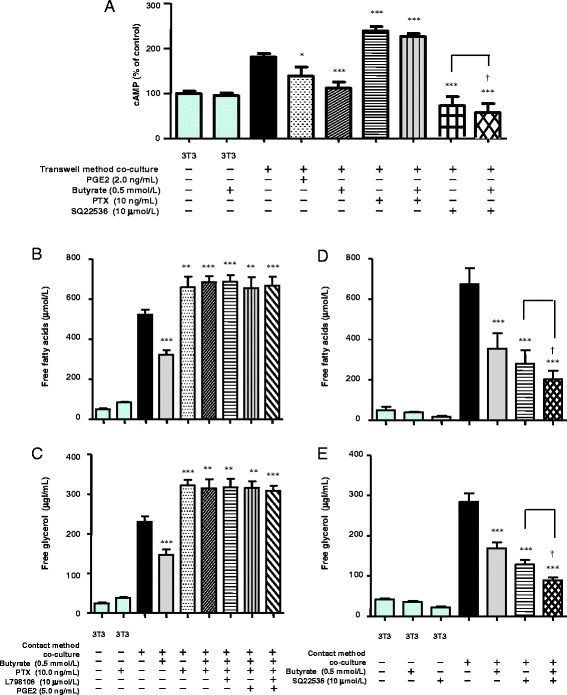
The effects of butyrate, PGE2, PTX, and adenylyl cyclase selective inhibitor (SQ22536) on cAMP formation in 3T3-L1 cells co-cultured using the transwell method for 24 h (**a**). The concentration of intracellular cAMP was measured using an assay kit. As the control, the concentration of intracellular cAMP was determined in untreated and non–co-cultured 3T3-L1 cells. The effects of butyrate, PGE2, PTX, and L798106 on lipolysis in cells co-cultured using the contact method for 24 h (**a**). Concentrations of FFAs (**b**) and free glycerol (**c**) in the co-culture medium were determined using an assay kit. As the control, the concentrations of FFAs and free glycerol were determined in the medium of untreated and non–co-cultured 3T3-L1 cells. The effects of butyrate and SQ22536 on lipolysis in cells co-cultured using the contact method for 24 h. Concentrations of FFAs (**d**) and free glycerol (**e**) in the co-culture medium were determined using an assay kit. As the control, the concentrations of FFAs and free glycerol were determined in the medium of untreated and non–co-cultured 3T3-L1 cells. Values from five independent experiments are expressed as the mean ± SD. *p < 0.05, **p < 0.01, ***p < 0.001 versus co-culture, as determined by ANOVA and the Tukey-Kramer test; ^†^ *p* < 0.05 for co-culture without butyrate and with SQ22536 versus co-culture with both butyrate and SQ22536, as determined by paired *t*-test

It is known that cAMP signaling via protein kinase A (PKA) is important for lipolysis [22]. To determine whether butyrate inhibits lipolysis by modulating cAMP levels, we examined the effects of PGE2 and butyrate on cAMP levels in 3T3-L1 cells co-cultured using the transwell method. As shown in Fig. 8a, cAMP levels significantly increased in 3T3-L1 cells co-cultured using the transwell method compared with 3T3-L1 cells cultured separately. Consistent with this result, lipolysis increased in 3T3-L1 cells co-cultured using the transwell method (Fig. 8b-e). PTX treatment increased cAMP above the levels observed in 3T3-L1 cells co-cultured using the transwell method (Fig. 8a) and increased lipolysis in cells co-cultured using the contact method (Fig. 8b, c). These results are also consistent with previously reported data [7]. Under treatment with PTX, we did not observe any additional effects of butyrate, L798106, and PGE2.

We also examined whether the adenylyl cyclase selective inhibitor SQ22536 suppresses cAMP production and lipolysis in co-cultured cells. As shown in Fig. 8a, SQ22536 completely suppressed cAMP production in 3T3-L1 cells co-cultured using the transwell method. SQ22536 inhibited the release of FFAs and free glycerol in cells co-cultured using the contact method by approximately 70% of the level observed in co-culture (Fig. 8d, e). Treatment with both SQ22536 and butyrate synergistically suppressed cAMP production and the release of FFAs and free glycerol. Thus, these results suggest that PGE2 and butyrate inhibit lipolysis by modulating cAMP levels.

**Fig. 9:**
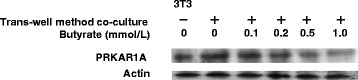
Effect of butyrate on the expression of PRKAR1A and actin proteins in 3T3-L1 cells co-cultured using the transwell method for 24 h. Expression of PRKAR1A and actin was detected by western blotting

Next, to determine whether butyrate reduces activation of PKA after decreasing cAMP, we examined the expression of PRKAR1A in 3T3-L1 cells. Butyrate treatment resulted in a dose-dependent decrease in PRKAR1A expression (Fig. 9).

### Effects of NF-κB- and COX2-selective inhibitors on lipolysis in TNF-α–stimulated 3T3-L1 cells

**Fig. 10:**
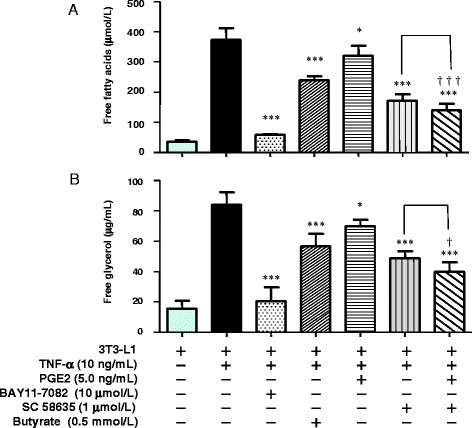
Effect of PGE2, butyrate, NF-κB–selective inhibitor (BAY11-7082), or COX2-selective inhibitor (SC 58635) on lipolysis in TNF-α–stimulated 3T3-L1 cells after 24 h. Concentrations of FFAs (**a**) and free glycerol (**b**) in the co-culture medium were determined using an assay kit. As the control, the concentrations of FFAs and free glycerol were determined in the medium of untreated and non–co-cultured 3T3-L1 cells. Values from five independent experiments are expressed as the mean ± SD. *p < 0.05 and ***p < 0.001 versus TNF-α–stimulated 3T3-L1 cells, as determined by ANOVA and the Tukey-Kramer test; ^†^ *p* <0.05, ^†††^ *p* <0.001 for TNF-α (+) and PGE2 (−) and SC 58635 (+) versus TNF-α (+) and PGE2 (+) and SC 58635 (+), as determined by paired *t*-test

TNF-α exerts a lipolytic effect via an NF-κB–mediated pathway in adipocytes [23, 24]. Previously, we showed that co-culture increases TNF-α production, whereas butyrate reduces the production of TNF-α in co-cultured cells and inhibits NF-κB activity in co-cultured macrophages, and we also demonstrated that either anti–TNF-α antibody or butyrate reduces the release of both FFAs and free glycerol from TNF-α–stimulated 3T3-L1 cells [7]. Here, we examined the importance of PGE2 in the effect of butyrate on lipolysis in TNF-α–stimulated 3T3-L1 cells. TNF-α enhanced FFAs and free glycerol release, and the NF-κB–selective inhibitor BAY11-7082 completely suppressed TNF-α–enhanced release of FFAs and free glycerol (Fig. 10a, b). Butyrate reduced the release of FFAs and free glycerol from TNF-α–stimulated 3T3-L1 cells by approximately 40% of the level of inhibition observed with BAY11-7082. Exogenous PGE2 reduced the release of FFAs and free glycerol by approximately 20% of the level of inhibition observed with BAY11-7082.

In contrast, the COX2-selective inhibitor SC 58635 reduced TNF-α–enhanced release of FFAs and free glycerol to a greater degree than butyrate. This result suggests that other COX2 metabolites in addition to PGE2 are associated with lipolysis.

### Effect of silent interference (si)RNAs targeting GPR41 or 109A on lipolysis in TNF-α–stimulated 3T3-L1 cells

**Fig. 11:**
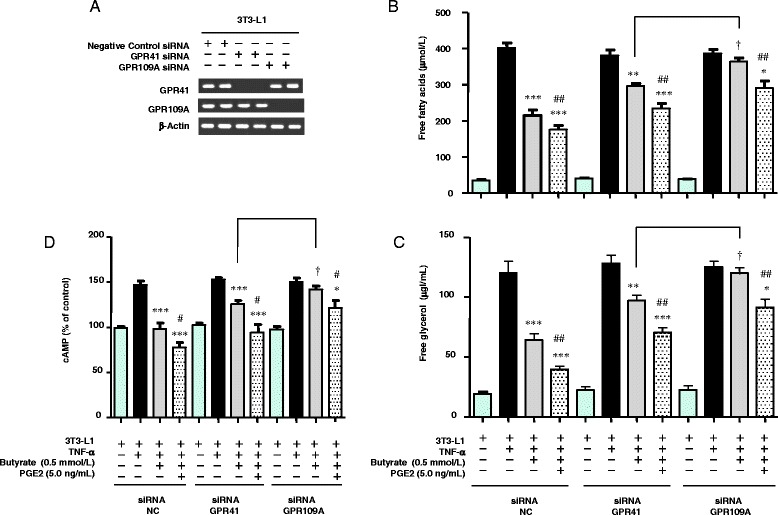
Treatment with NC, GPR41, and 109A siRNA and detection of the expression of GPR41 and 109A RNA in 3T3-L1 cells (**a**). Effect of butyrate, PGE2, and NC, GPR41, and 109A siRNA treatment on lipolysis assayed in TNF-α–stimulated 3T3-L1 cells after 24 h. Concentrations of FFAs (**b**) and free glycerol (**c**) in the medium were determined using an assay kit. As the control, the concentrations of FFAs and free glycerol were determined in the medium of untreated 3T3-L1 cells. The effects of butyrate, PGE2, and NC, GPR41, and 109A siRNA treatment on cAMP accumulation in TNF-α–stimulated 3T3-L1 cells after 24 h (**d**). The concentration of intracellular cAMP was measured using an assay kit. As the control, the concentration of intracellular cAMP was determined in untreated 3T3-L1 cells. Values from five independent experiments are expressed as the mean ± SD. *p < 0.05, **p < 0.01, ***p < 0.001 versus TNF-α–stimulated 3T3-L1 cells, as determined by ANOVA and the Tukey-Kramer test; and ^#^ *p* < 0.05 and ^##^ *p* < 0.01 versus TNF-α–stimulated 3T3-L1 cells treated with butyrate (0.5 mmol/L), as determined by ANOVA and the Tukey-Kramer test; ^†^ *p* < 0.05 for TNF-α–stimulated 3T3-L1 cells treated with GPR41 siRNA and butyrate (+) versus 3T3-L1 cells treated with GPR109A siRNA and butyrate (+), as determined by paired *t*-test

The results shown in Fig. 2 suggest that butyrate stimulates PGE2 production via GPR41- and/or GPR109A-mediated pathways. Here, we examined which is the principal pathway regulating the effect of butyrate on lipolysis using TNF-α–stimulated 3T3-L1 cells. Using siRNAs targeting either GPR41 or GPR109A, we blunted the expression of GPR41 or GPR109A, respectively (Fig. 11a). Using siRNA targeting GPR41, the inhibitory effect of butyrate on the release of FFAs and glycerol was reduced by half (Fig. 11b, c). Using siRNA targeting GPR109A, the inhibitory effect of butyrate on the release of FFAs and free glycerol was almost completely abrogated (Fig. 11b, c). As shown in Fig. 11d, cAMP levels significantly increased in TNF-α–stimulated 3T3-L1 cells. Butyrate reduced cAMP levels, and exogenous PGE2 further reduced the cAMP levels. These results suggest that PGE2 inhibits lipolysis through a mechanism involving both butyrate-related and non-butyrate–related pathways. Treatment with siRNA targeting GPR109A attenuated the inhibitory effect of butyrate on cAMP levels to a greater extent than treatment with siRNA targeting GPR41 (Fig. 11d). These results correspond to the results of Fig. 11b and c. Thus, the GPR109A-mediated pathway may predominate, but the GPR41-mediated pathway also plays a role in regulating the effect of butyrate on lipolysis in TNF-α–stimulated 3T3-L1 cells.

In the case of both control cells and GPR41 or GPR109A knockdown cells, exogenous PGE2 caused a further significant reduction in the release of FFAs and free glycerol compared with butyrate. These results, in conjunction with those shown in Fig. 10, suggest that PGE2 inhibits lipolysis in TNF-α–stimulated 3T3-L1 cells via a mechanism involving both butyrate-mediated and non-butyrate–mediated pathways, although exogenous PGE2 had no significant additional suppressive effect on butyrate-treated cells co-cultured using the contact method (Fig. 7a, b).

## Discussion

In this study, we clarified the mechanism underlying the anti-lipolytic effect of butyrate in the interaction between macrophages and adipocytes. Butyrate up-regulated cPLA2 activity in co-cultured macrophages and COX2 expression in co-cultured macrophages and adipocytes, resulting in enhanced production of PGE2 and subsequent reductions in the levels of cAMP and PRKAR1A, leading to suppression of lipolysis.

We first examined the levels of PGE2 in the medium of cells co-cultured using both the contact and transwell methods. PGE2 levels increased markedly in the medium of cells co-cultured using the contact method, whereas PGE2 levels were very low in the medium of cells cultured separately. Interestingly, an additional dose-dependent increase in PGE2 production was observed when cells were treated with butyrate at concentrations up to 0.5 mmol/L (Fig. 1a). The peak level of PGE2 production was higher when cells were co-cultured using the contact method than when they were cultured using the transwell method (Fig. 1b). Both cell types produced PGE2, and under co-culture conditions, butyrate stimulated PGE2 production, particularly in macrophages (Fig. 1c, d). These results suggest that in addition to the effects of other stimulators such as cytokines, direct cell-to-cell contact may play a significant role in PGE2 production. These data were similar to our previously reported data regarding TNF-α, MCP-1, and IL-6 production [7]. Furthermore, Iyer et al. reported that PGE2 concentrations in plasma and white adipose tissues are elevated in obese rats fed a high-carbohydrate/high-fat diet compared with cornstarch-fed normal rats and that increased PGE2 concentrations are correlated with increased adiposity [25]. Our results appear to support their data.

In this study, we chose the transwell method for the separate culture of RAW264.7 and 3T3-L1 cells. Using this approach, we found that cPLA2 activity in macrophages, sPLA2 activity in adipocytes and macrophages, and the expression of AdPLA2 protein and mRNA in adipocytes were up-regulated in co-culture, and butyrate treatment elevated cPLA2 activity to a greater degree in macrophages (Figs. 4 and 5). The observation that PGE2 was produced primarily by macrophages was consistent with those results. Butyrate may enhance interactions between cPLA2, COX2, and PGE2 in co-cultured macrophages.

Butyrate modulates gene transcription by inducing hyperacetylation of histones through inhibition of histone deacetylase [20]. Butyrate combined with lipopolysaccharide synergistically increases COX2 gene expression in macrophages through both acetylation and phosphorylation of histone H3 at the promoter site [26]. Consistent with previous reports [16, 17], in the present study, butyrate increased the expression of both COX2 protein and mRNA in co-cultured adipocytes and macrophages (Fig. 3). Although we did not examine detailed mechanisms in both cell types, we hypothesize that butyrate treatment increases COX2 gene and protein expression in both adipocytes and macrophages by inhibiting HDAC. Furthermore, TSA mimicked the up-regulating effect of butyrate on PGE2 production (Fig. 6). Although our results did not provide direct evidence, taken together, they suggest that the inhibition of HDAC is a mechanism by which butyrate increases the production of PGE2.

Co-culture with macrophages increased the release of FFAs and free glycerol from adipocytes into the culture medium, and butyrate suppressed this release. PGE2 also suppressed the release of FFAs and free glycerol, although to a lesser extent than butyrate. Complementarily, the EP3-selective antagonist L798106 attenuated the PGE2- and butyrate-dependent inhibition of lipolysis (Fig. 7). We determined that approximately 40% of the suppressive effect of butyrate depends on the PGE2-mediated pathway, whereas 60% depends on the non-PGE2–mediated pathway.

TNF-α exerts a lipolytic effect via an NF-κB–mediated pathway in adipocytes [23, 24]. SCFAs inhibit the activation of NF-κB, leading to suppression of the multiple responses in both immune cells and various other cell types [27]. Previously, we showed that co-culture increases TNF-α production, whereas butyrate decreases TNF-α production by reducing TNF-α mRNA expression in co-cultured both cells and inhibiting NF-κB activity in co-cultured macrophages [7]. We thus speculated that butyrate reduces lipolysis via a PGE2-mediated pathway by suppressing the production of TNF-α. In this study, we examined the importance of PGE2 in regulating the effect of butyrate on lipolysis in TNF-α–stimulated 3T3-L1 cells (Fig. 10). Butyrate reduced the release of FFAs and free glycerol by approximately 40% of the level of inhibition observed with the NF-κB–selective inhibitor, BAY11-7082; exogenous PGE2 reduced the release of FFAs and free glycerol by approximately 20% of that level. Taken together, these results suggest that butyrate contributes at least in part to the effect on lipolysis via an NF-κB–mediated pathway and that additional non-PGE2 mechanisms also appear to be relevant.

The COX2-selective inhibitor SC 58635 reduced NF-κB–mediated lipolysis to a greater degree than butyrate, suggesting that the mechanism involves other COX2 metabolites in addition to PGE2 and butyrate (Fig. 10).

Previously, we showed that butyrate significantly reduces FFAs release in TNF-α–stimulated 3T3-L1 cells by diminishing total lipase activity by suppressing the expression of ATGL and the phosphorylation of HSL (pSer^660^) in co-cultured 3T3-L1 cells [7]. Exogenous TNF-α induces an increase in intracellular cAMP production in adipocytes [28]. Elevation of intracellular cAMP activates cAMP-dependent protein kinase, with subsequent expression of ATGL and phosphorylation of HSL in adipocytes [29, 30]. PGE2 down-regulates lipolysis by inhibiting cAMP production in adipocytes [10, 21]. To further elucidate the underlying mechanism by which butyrate inhibits lipolysis in adipocytes, we therefore examined the effects of PGE2 and butyrate on cAMP levels in co-cultured 3T3-L1 cells. We found that cAMP levels were significantly higher in co-cultured 3T3-L1 cells compared with 3T3-L1 cells cultured separately, and butyrate treatment restored cAMP levels, lipolysis, and PRKAR1A expression (Figs. 8 and 9). Thus, the present data appear to support our previous results. It is unclear why PTX increased cAMP levels to a greater degree than co-culture in adipocytes and why treatment with both SQ22536 and butyrate synergistically suppressed cAMP levels and lipolysis. There may be other mechanisms regulating cAMP levels in adipocytes.

GPR41, GPR43, and GPR109A exhibit affinity for SCFAs [19, 31–33]. PTX, which is known to inactivate Gi/o proteins, negates the effect of GPR41 and GPR109A but not that of GPR43 [33, 34]. The observation that PTX completely reversed the up-regulated production of PGE2 induced by butyrate (Fig. 2) suggests that the effect of butyrate on PGE2 production may involve GPR41- and/or GPR109A-mediated signaling. We therefore sought to determine which pathway plays the greatest role in regulating the effect of butyrate on lipolysis in TNF-α–stimulated 3T3-L1 cells. Using siRNAs targeting GPR41 or GPR109A, we found that the GPR109A-mediated pathway predominates, but the GPR41-mediated pathway also plays a role in regulating the effect of butyrate on lipolysis in TNF-α–stimulated 3T3-L1 cells. Interestingly, inflammation stimulates GPR109A expression in adipocytes and macrophages [35]. TNF-α also enhances GPR109A expression in adipocytes, leading to an acceleration of the suppressive effect of butyrate on lipolysis.

**Fig. 12:**
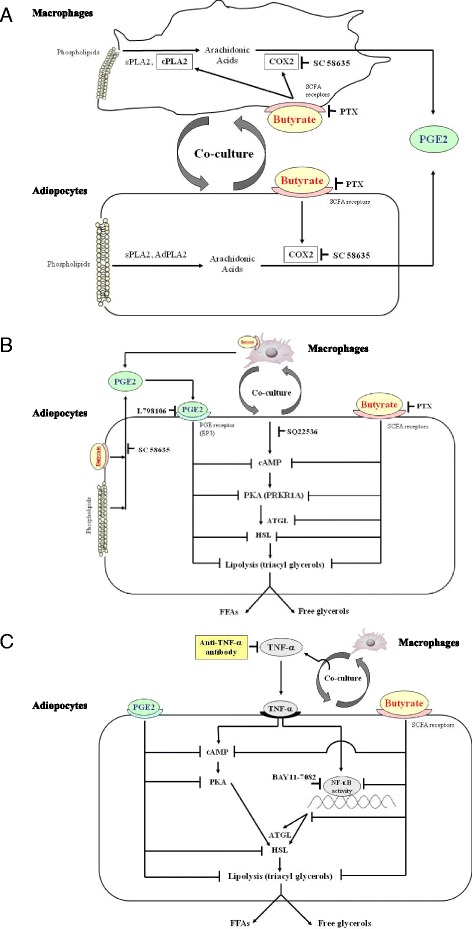
Hypothetical pathways based on the results of the present study and our previous data. **a** The effect of butyrate on PGE2 production in the interaction between co-cultured macrophages and adipocytes. Co-culture elevates cPLA2 activity in macrophages, sPLA2 activity in adipocytes and macrophages, and the expression of AdPLA2 protein and mRNA in adipocytes. Butyrate elevates cPLA2 activity to a greater degree in macrophages. Co-culture elevates COX2 expression in both cells, and butyrate further enhances COX2 expression in both cells. Thus, butyrate increases PGE2 production more than co-culture alone. **b** The effects of butyrate and PGE2 on lipolysis in co-cultured adipocytes. Co-culture increases cAMP and PKA (PRKAR1A) levels in adipocytes and increases the release of FFAs and free glycerol into the medium (lipolysis). Butyrate suppresses cAMP and PKA levels, and exogenous PGE2 has a lesser effect than butyrate. These suppressive effects may reduce the activities of lipases, including ATGL and HSL, thus resulting in inhibition of lipolysis. **c** The effects of butyrate and exogenous PGE2 on cAMP- and NF-κB–mediated lipolysis in TNF-α–stimulated 3T3-L1 adipocytes. Co-culture increases TNF-α production. TNF-α increases cAMP, leading to increased lipolysis. Anti–TNF-α antibody, butyrate, or exogenous PGE2 decrease cAMP levels and reduce lipolysis in TNF-α–stimulated 3T3-L1 cells. The GPR109A-mediated pathway may be the predominant pathway regulating the effect of butyrate on lipolysis in TNF-α–stimulated 3T3-L1 cells

Several hypothetical pathways are shown in Fig. 12. Co-culture elevates PGE2; butyrate increases PGE2 levels further by elevating cPLA2 activity in macrophages and COX2 expression in both types of cells in co-culture (Fig. 12a). Co-culture also increases cAMP and PKA levels in adipocytes and increases the release of FFAs and free glycerol into the medium (lipolysis). Butyrate suppresses cAMP and PKA levels, and exogenous PGE2 has a lesser effect in this regard than butyrate. These suppressive effects may reduce the activities of lipases, including ATGL and HSL, resulting in inhibition of lipolysis (Fig. 12b). Co-culture increases TNF-α production. TNF-α in turn increases cAMP levels, leading to increased lipolysis. Anti–TNF-α antibody, butyrate, or exogenous PGE2 decrease cAMP levels and reduce lipolysis in TNF-α–stimulated 3T3-L1 cells. The GPR109A-mediated pathway thus appears to predominate in regulating the effect of butyrate on lipolysis in TNF-α–stimulated 3T3-L1 cells (Fig. 12c).

Rumberger et al. recently reported that butyrate increases lipolysis in 3T3-L1 cells [36]. However, their methods differed from ours in several respects. First, they used 3T3-L1 cells but not co-culture. In addition, they used a higher concentration of butyrate than we did (5 mmol/L versus 0.1–1.0 mmol/L). Finally, they used cells at 3–10 days post-differentiation, whereas we used cells at 20 days post-differentiation. Similarly, in this study, we found that PGE2 has a small additional effect on TNF-α–stimulated 3T3-L1 cells compared with butyrate, but this effect was not observed with 3T3-L1 cells co-cultured with RAW264.7 cells. Thus, differences in the condition of the cells could have affected the action of butyrate. To fully elucidate the suppressive effect of butyrate on lipolysis in adipose tissues, therefore, further in vitro and in vivo studies will be necessary.

Finally, many other nutritional supplements, such as other SCFAs, glucose, or fructose, may regulate lipolysis in 3T3-L1 cells. Further investigations are thus needed to elucidate the interactions between adipocytes and macrophages in metabolic disorders.

## Conclusions

Butyrate attenuates lipolysis in adipocytes co-cultured with macrophages via non-PGE2–mediated and PGE2-mediated pathways.

## Methods

### Reagents and cells

Butyric acid and FFA-free bovine serum albumin (BSA) were purchased from Wako Chemicals (Osaka, Japan). All other chemicals were obtained from Nacalai Tesque (Kyoto, Japan). RAW264.7 mouse macrophages and 3T3-L1 mouse pre-adipocytes (American Type Culture Collection, Manassas, VA) were cultured in DMEM (Nissui Pharmaceutical Co., Ltd., Tokyo, Japan) with 292 μg/mL L-glutamine, 100 units/mL penicillin, 100 μg/mL streptomycin, and 10% fetal bovine serum (FBS) at 37 °C under 5% CO_2_. Differentiation of 3T3-L1 pre-adipocytes was induced by addition of 0.5 mmol/L 13-isobutyl-1-methylxanthine, 2.5 μmol/L dexamethasone, and 10 μg/mL insulin (Wako Chemical) in DMEM containing 10% FBS, beginning 2 days after the cells reached confluence in a 24-well plate. The medium was then replaced with DMEM containing 10% FBS and 5.0 μg/mL insulin and was changed every 2 or 3 days. Twenty days after induction of differentiation, the hypertrophied 3T3-L1 adipocytes were used in experiments. Cell viability was assessed using an MTT cell viability assay kit (R&D Systems, Minneapolis, MN).

### Co-culture of adipocytes and macrophages

Adipocytes and macrophages were co-cultured using two different methods, the contact method and the transwell method [6, 7]. In the contact method, serum-starved differentiated 3T3-L1 cells (5.0 × 10^5^ cells/well) were cultured in a 24-well plate, and RAW264.7 cells (2.0 × 10^5^ cells/well) were then plated onto the 3T3-L1 cells. The cells were cultured in contact with each other for 24 h in the presence of butyrate (0, 0.1, 0.2, 0.5, or 1.0 mmol/L) in 2% FFA-free BSA DMEM and then harvested. In the transwell method, cells were co-cultured using 24-well transwell plate inserts with a 0.4-μm porous membrane (Corning, New York, NY), which separated the serum-starved 3T3-L1 cells (5.0 × 10^5^ cells/well) in the lower chamber from the serum-starved RAW264.7 (5.0 × 10^5^ cells/well) cells in the upper chamber. After incubation with butyrate (0, 0.1, 0.2, 0.5, or 1.0 mmol/L) in 2% FFA-free BSA for 24 h, both the RAW264.7 cells in the upper chamber and the 3T3-L1 cells in the lower chamber were harvested. We used the contact method for experiments with culture medium, whereas we used the transwell method for experiments with each cell type separately, such as for the collection of mRNA or detection of cAMP, according to our previous report [7].

### Measurement of PGE2

The concentration of PGE2 in the culture medium was determined using a Prostaglandin E2 Express EIA kit (Cayman Chemical Co., Ann Arbor, MI) in accordance with the manufacturer’s instructions. Levels of PGE2 secreted by RAW264.7 or 3T3-L1 cells cultured separately were determined as a control.

### 3T3-L1 and RAW264.7 CM

Hypertrophied 3T3-L1 adipocytes were cultured in 2% FFA-free BSA in a 24-well plate for 24 h, after which the supernatant was collected as 3T3-CM and stored at −80 °C until use. RAW264.7 macrophages were cultured in 2% FFA-free BSA in a 24-well plate for 24 h, after which the supernatant was collected as RAW-CM and stored at −80 °C until use. After incubation of 3T3-L1 cells with RAW-CM or incubation of RAW264.7 cells with 3T3-CM for 24 h with or without 0.5 mmol/L butyrate, PGE2 produced by both types of cells was measured.

### Effect of TSA on PGE2 production in co-culture

Cells were cultured using the contact method for 24 h in the presence of TSA (Cayman Chemical) (0, 25, 50, 100 nmol/L) in 2% FFA-free BSA culture medium and then harvested. TSA was dissolved in 0.5% (*v/v*) dimethylsulfoxide (DMSO).

### Effect of treatment with PTX

Using the contact method, 3T3-L1 adipocytes were pretreated with 10 ng/mL of PTX at 37 °C for 60 min. The cells were then cultured for 24 h in the presence of PTX and butyrate (0, 0.1, 0.2, 0.5, or 1.0 mmol/L) in 2% FFA-free BSA culture medium and then harvested.

### Lipolysis assay

Differentiated 3T3-L1 cells were incubated overnight using the contact method in serum-free DMEM with 2% FFA-free BSA. The cells were then co-cultured with RAW264.7 cells in the same medium for 24 h with butyrate (0, 0.5 mmol/L) and PGE2 (0, 5 ng/mL), after which the culture medium was harvested. The levels of FFAs in the co-culture medium were measured using an acyl-coenzyme A oxidase–based colorimetric assay method, which enables detection of C8 (octane) and longer-chain fatty acids. The assay was conducted in accordance with the manufacturer’s instructions [37]. Levels of free glycerol in the medium were measured using an Adipolysis Assay kit (Cayman Chemical). To examine the effects of BAY 11-7082 and SC 58635, 3T3-L1 cells were pretreated by culturing at 37 °C for 60 min in serum-free DMEM with 0.5% (*v/v*) DMSO (vehicle), 5.0 ng/mL PGE2 (Cayman Chemical) dissolved in DMSO, 10 μmol/L BAY 11-7082 (Santa Cruz Biotechnology, Santa Cruz, CA) dissolved in DMSO, or 1 μmol/L SC 58635 (R&D Systems) dissolved in DMSO. 3T3-L1 cells were then stimulated with TNF-α (with or without butyrate) in 2% FFA-free BSA medium and cultured for 24 h, after which levels of FFAs and free glycerol released into the medium were measured.

### Quantitative reverse transcription–polymerase chain reaction (qRT-PCR)

Total RNA was extracted from RAW264.7 cells in the transwell plate upper chamber and from 3T3-L1 cells in the lower chamber using TRIzol reagent (Invitrogen, Carlsbad, CA). Single-stranded complementary DNA (cDNA) was synthesized from 1 μg of total RNA using a ReverTraAce-α first-strand cDNA synthesis kit (Toyobo, Osaka, Japan). Samples were then incubated at 37 °C for 60 min. The temperature of the reaction was then raised to 94 °C for 5 min in order to inactivate the enzyme, after which the temperature was reduced to 4 °C. cDNAs were synthesized using a Gene Amp PCR System 9700 (Applied Biosystems, Carlsbad, CA). To quantify mRNA expression, real-time PCR was performed using a LightCycler System (Roche Diagnostics, Mannheim, Germany) and LightCycler Fast Start DNA Master Plus SYBR Green 1 (Roche Diagnostics), as described previously [7]. The linearity of amplification was determined for COX2 and β-actin cDNAs from RAW264.7 or 3T3-L1 cells and for pla2g16 (AdPLA) cDNAs from 3T3-L1 cells. Primers for the different genes are listed in Table 1. The real-time PCR program was as follows: for COX2, AdPLA, and β-actin, 45 cycles of 95 °C for 10 s, 55 °C for 10 s, and 72 °C for 10 s; for LPL, 45 cycles of 95 °C for 10 s, 60 °C for 10 s, and 72 °C for 10 s. The level of β-actin mRNA was adopted as an internal standard for the determination of target mRNA levels. Samples were analyzed five times. Ct values (the cycle at which the emitted fluorescence signal was significantly higher than the background level and inversely proportional to the initial template copy number) were calculated by the LightCycler software. Finally, the relative expression level of each gene was calculated according to the 2^-ΔΔCt^ method.

**Table 1:** Primer sequences used for qRT-PCR or RT-PCR

Gene	Forward 5′ → 3′	Reverse 5′ → 3	GeneBank accesion number
COX2	GGGAAGCCTTCTCCAACC	GAACCCAGGTCCTCGCTT	NM_011198.3
pla2g16 (AdPLA)	TACAGGCTGACCAGCGAGAACT	CCACTCCAGCGATGCCTACCG	BC024581.1
GPR41	TCCGTCTACCTGTTGGTGTTCC	CATCTCTAGTCGTACAGGCAGG	NM_001033316.2
GPR109A	TTCTCCTGATCATCTGCCTGCC	TGTCTGTCCATCTGTCTCTGCC	NM_030701.3
β-Actin	CTAAGGCCAACCGTGAAAAG	ACCAGAGGCATACAGGGACA	NM_007393.3

*COX2* cyclooxygenase-2, *AdPLA* adipose specific PLA2, *LPL* lipoprotein lipase

### Reverse transcription-polymerase chain reaction (RT-PCR)

Total RNA was extracted from the hypertrophied 3T3-L1 adipocytes in 24 well plate using TRIzol reagent (Invitrogen). Single-stranded cDNA was synthesized from 1 μg of total RNA using a ReverTra Ace-α, first strand cDNA synthesis kit (Toyobo). Incubation was carried out at 37 °C for 60 min. The temperature of the reaction was then raised to 94 °C for 5 min in order to inactivate the enzyme and was then reduced to 4 °C. To detetect GPR41, GPR109A and β-actin mRNA expression, PCR amplification was performed using a Gene Amp PCR System 9700 (Applied Biosystems). Primers for the different genes are listed in Table 1.

### RNA interference

Mouse GPR41 siRNA duplex and negative control (NC) siRNA were purchased from Santa Cruz Biotechnology. Mouse GPR109A siRNA duplexes were purchased from Origene Technologies (Rockville, MD). Hypertrophied 3T3-L1 adipocytes were seeded in 24-well plates and transfected with each targeting siRNA at 10 μmol/L (final concentration 5 pmol/well). The siRNA and lipofectamine RNA iMAX reagent (Invitrogen) were diluted separately in Opti-MEM Medium (Invitrogen), then the two solutions were gently mixed and incubated for 5 min at room temperature. 3T3-L1 cells were transfected with siRNA-lipofectamine complexes and incubated for 48 h at 37 °C under 5% CO_2_ and then used in the experiments.

### Western blotting

Cells were co-cultured using the transwell method. Protein was extracted from RAW264.7 cells cultured in the upper chamber and from 3T3-L1 cells cultured in the lower chamber. For western blot analysis, cells were lysed using M-PER Mammalian Protein Extraction reagent (Pierce, Rockford, IL) with a protease and phosphatase inhibitor cocktail (Nacalai Tesque). Protein concentration was determined using a protein assay reagent (Bio-Rad Laboratories, Hercules, CA). Denatured proteins were separated using SDS-polyacrylamide gel electrophoresis on a 10% polyacrylamide gel and then transferred onto Immobilon-P membranes (Millipore, Billerica, MA).

The following antibodies were used: rabbit anti-mouse HRASLA3 (AdPLA) (1:1000; BD Biosciences, Franklin Lakes, NJ), rabbit anti-mouse COX2 (1:1000; Santa Cruz Biotechnology), rabbit anti-mouse PRKAR1A (1:1000; GeneTex, Irvine, CA), rabbit anti-mouse actin (1:1000; Santa Cruz Biotechnology), and anti-rabbit horseradish peroxidase–conjugated immunoglobulin G (1:2000; GE Healthcare, Bucks, UK). The blots were developed using ECL (GE Healthcare). Relative protein expression levels were determined by dividing the band intensity of the product of interest by that of the actin control band. The intensity of the level of objective protein expression was analyzed using ImageJ software (http://imagej.nih.gov/ij/) [38].

### Calcium-dependent cPLA2 and sPLA2 activity

Protein was extracted from 3T3-L1 cells in the lower chamber and RAW264.7 cells in the upper chamber of the transwell plate. Cell lysates were prepared after 24 h of co-culture with butyrate treatment. The protein concentration was adjusted to 1 mg/mL and cPLA2 and sPLA2 activity was assayed five times using cPLA2 and sPLA2 colorimetric assay kit (Cayman Chemical), respectively, according to the manufacturer’s instructions.

### Measurement of intracellular cAMP concentration

Differentiated 3T3-L1 cells cultured using the transwell method were treated with butyrate. The intracellular cAMP concentration was determined using a cAMP Total EIA kit (Arbor Assay Co., Ann Arbor, MI) in accordance with the manufacturer’s instructions. The results were standardized by dividing the cAMP concentration by the intracellular total protein concentration (1 mg/mL).

### Effect of butyrate and EP3 antagonist on lipolysis in co-culture

Differentiated 3T3-L1 cells were pretreated for 1 h with 0.5% (*v/v*) DMSO alone (vehicle), 10 μmol/L of the EP3-selective antagonist L798106 (Sigma-Aldrich, St. Louis, MO), or 5.0 ng/mL of PGE2 dissolved in DMSO and diluted in serum-free DMEM. The cells were then co-cultured for 24 h using the contact method in DMEM with 2% FFA-free BSA. Cells were also co-cultured in the same manner with 0 and 0.5 mmol/L butyrate and 0 and 5.0 ng/mL PGE2. Concentrations of FFAs in the co-culture medium were measured using an acyl-coenzyme A oxidase–based colorimetric assay kit, and free glycerol levels were measured using an Adipolysis Assay kit.

### Effect of butyrate on cAMP levels in 3T3-L1 cells

Differentiated 3T3-L1 cells were pretreated for 1 h with 0.5% (*v/v*) DMSO alone (vehicle) or 10 μmol/L of the adenylyl cyclase selective inhibitor SQ22536 (Abcam, Cambridge, UK) dissolved in DMSO and diluted in serum-free DMEM. Differentiated 3T3-L1 cells cultured using the transwell method were treated with butyrate, PGE2, PTX, or SQ22536 for 24 h. Protein was extracted from 3T3-L1 cells cultured in the lower chamber, and cAMP levels were determined using a cAMP Total EIA kit in accordance with the manufacturer’s instructions. The results were standardized by dividing the cAMP concentration by the intracellular total protein concentration (1 mg/mL).

### Effect of butyrate and an adenylyl cyclase selective inhibitor on lipolysis in co-cultured cells

Differentiated 3T3-L1 cells were pretreated for 1 h with 0.5% (*v/v*) DMSO alone (vehicle) or 10 μmol/L SQ22536 dissolved in DMSO and diluted in serum-free DMEM. The cells were then co-cultured for 24 h using the contact method in DMEM with 2% FFA-free BSA and treated with butyrate or SQ22536 in 24 h. Concentrations of FFAs in the co-culture medium were measured using an acyl-coenzyme A oxidase–based colorimetric assay kit, and free glycerol levels were measured using an Adipolysis Assay kit.

### Statistical analysis

Data are expressed as mean ± SD. We used a paired *t*-test for comparisons between two groups or ANOVA with a Tukey-Kramer post-hoc test for comparisons between more than three groups. Statistical analyses were performed using GraphPad Prism software, ver. 5.0 (GraphPad Software Inc., San Diego, CA). A *p* value <0.05 in two-tailed tests was considered to indicate statistical significance.

## Abbreviations

AdPLA: Adipose-specific PLA2
ATGL: Adipose triglyceride lipase
BSA: Bovine serum albumin
butyrate: Sodium butyrate
cAMP: Cyclic adenosine monophosphate
c-DNA: Complementary DNA
COX2: Cyclooxygenase-2
cPLA2: Calcium-dependent cytosolic phospholipase A2
DMSO: Dimethylsulfoxide
ELISA: Enzyme-linked immunosorbent assay
EP3: Prostaglandin E receptor 3
FBS: Fetal bovine serum
FFAs: Free fatty acids
GPR: G-protein–coupled receptor
HSL: Hormone-sensitive lipase
IL: Interleukin
MCP-1: Monocyte chemoattractant protein-1
NF-κB: Nuclear factor-kappa B
PGE2: Prostaglandin E2
PLA2: Phospholipase A2
PRKAR1A: Protein kinase A type 1-α regulatory subunit
PTX: Pertussis toxin
qRT-PCR: Quantitative reverse transcription–polymerase chain reaction
SCFAs: Short-chain fatty acids
siRNA: Small interfering RNA
sPLA2: Secretory phospholipase A2
TNF-α: Tumor necrosis factor-α
TSA: Trichostatin A
